# Relationship Between Gender and 1-Year Mortality in ANCA-Associated Vasculitis Patients: A Single-Center Retrospective Analysis and Meta-Analysis

**DOI:** 10.3389/fmed.2022.945011

**Published:** 2022-07-13

**Authors:** Qing Zhu, Fen Li, Xi Xie, Bilin Chen, Qianwen Yu, Yusong Wei, Yan Ge

**Affiliations:** ^1^Department of Rheumatology and Immunology, The Second Xiangya Hospital, Central South University, Changsha, China; ^2^Department of Rheumatology and Immunology, Jingzhou Hospital, Yangtze University, Jingzhou, China

**Keywords:** gender, 1-year mortality ratio, ANCA-associated vasculitis (AAV), prognosis, meta-analysis

## Abstract

**Objective:**

The relationship between gender and short-term prognosis of patients with anti-neutrophil cytoplasmic antibody (ANCA) associated vasculitis (AAV) is unclear, hence single-center retrospective analysis and meta-analysis were conducted to determine the relationship.

**Methods:**

Initially treated patients with AAV were retrospectively enrolled. Data of clinical manifestation, laboratory indicators, Birmingham vasculitis activity score (BVAS), therapeutic treatments, and the patients' situations within 1 year were recorded. First, we compared the basic characteristics between male and female patients. Second, the risk factors associated with a 1-year mortality rate of patients with AAV were evaluated. Finally, a meta-analysis was performed to explore the effect of gender on 1-year mortality in patients with AAV.

**Results:**

The study involved 84 patients with AAV, including 33 female and 51 male participants. In total, 14 people died (12 males and 2 females) and 70 survived in the 1st year. Statistical differences were noted in the age of onset, the course of the disease, WBC, HB, N, ESR, CRP, BUN, ALT and ALB, BVAS, and 1-year mortality rate between male and female participants. In male patients, elevated Scr, NLR, PLT, and RDW-CV were associated with poor AAV (*P* < 0.05) prognosis. The meta-analysis verified that male gender was an independent risk factor for the 1-year mortality of patients with AAV(OR = 1.54).

**Conclusion:**

Significant sex-specific differences were found in patients with AAV. Male patients contributed to 1.54-fold of 1-year mortality risk in patients with AAV by meta-analysis. More attention should be paid to the mortality risk of male patients with AAV in the early stage.

## Introduction

Anti-neutrophil cytoplasmic antibody (ANCA) associated vasculitis (AAV) is a rare and severe systemic necrotizing small-vessel vasculitis (1) characterized by vascular inflammation, endothelial injury, and tissue damage (2). AAV is generally accompanied by the presence of ANCA in serum. ANCA is a serum auto-antibody for proteins present in neutrophils, which is a serological marker for small vessel vasculitis (3). The two major antigens of ANCA are myeloperoxidase (MPO) and proteinase 3 (PR3) (4). This distinctly pauci-immune form of vasculitis includes three clinic-pathological types: microscopic polyangiitis (MPA), granulomatosis with polyangiitis (GPA), and eosinophilic granulomatosis with polyangiitis (EGPA) (5). The characteristic histology of MPA shows a necrotizing small-vessel vasculitis with little or absent immune deposits (pauci-immune vasculitis) and the absence of necrotizing granulomas (6, 7). GPA is mainly characterized by a typical histological triad, including granulomatous inflammation with local necrosis and necrotizing small-vessel vasculitis (8, 9). EGPA is a systemic small-vessel vasculitis associated with asthma, eosinophilia, and neuropathy. Pathologically, EGPA is considered to be a triad consisting of necrotizing vasculitis, eosinophilic infiltration, and extravascular granuloma, and the presence of ANCAs is associated with the clinical and pathological features of eosinophilic granulomatosis (10, 11). MPO–ANCA is often detected in the sera of patients with MPA and EGPA, while, PR3-ANCA is a useful marker for GPA. The clinical manifestations of AAV are roughly variable, and the gold standard for diagnosis is tissue biopsy (12). Although the etiology and pathogenesis of AAV are complex, genetic factors play a certain role which to some extent explained the geographical differences (5). The disease is more common in white and Asian populations and less common in African–American populations (12, 13).

Patients with AAV have a 1-year mortality rate of up to 80% in the natural disease course they do not receive treatment (14), and even with intensive treatment, patients still carry a 2.7-fold increased risk of death compared with the general population (15, 16). Although several studies have shown that the predictors of poor prognosis for patient survival were dialysis dependency or high creatinine level at initial diagnosis, high Birmingham vasculitis activity score (BVAS), and older age (17–19), the specific mechanism remains unknown. Recent studies had specifically focused on the association between gender and the prognosis of AAV. It was reported that male gender was an independent risk factor for all-cause mortality of patients with AAV during long-term follow-up (20); however, whether male gender was related to their short-term mortality (1-year mortality) had no unified conclusion. Among Caucasians, male patients had an increased risk of death and a higher mortality rate within 28 days of ICU admission compared with female patients (21). Male sex was also associated with an increased risk of end-stage renal disease (ESRD) (22). However, in 2007 Abe et al. found that gender was not associated with the prognosis of AAV (19). Considering there have been few studies about the relationship between gender and 1-year mortality of patients with AAV and since there were no data from mainland China, we conducted retrospective research on inpatients in our hospital and a meta-analysis to determine the impact of gender on the 1-year mortality ratio of patients with AAV. We hoped to find some correlation between gender and 1-year mortality in patients with AAV to provide a predictive index for clinicians.

## Materials and Methods

### Single-Center Retrospective Analysis

#### Patients Enrolled

Patients with AAV admitted and first diagnosed in the Second Xiangya Hospital of Central South University between January 2014 to December 2019 were retrospectively enrolled. All patients diagnosed with AAV met the diagnostic criteria established in the 2012 Chapel Hill Consensus Conference (CHCC) (6), and those with overlap syndrome or secondary vasculitis, or severe chronic diseases such as a malignant tumor, hypertension, and diabetes were excluded. Cases lost to follow-up were also excluded. This study was approved by the Ethics Committee of the Second Xiangya Hospital of Central South University and was in accordance with the Declaration of Helsinki. This study had no adverse influence on the rights or welfare of patients. Informed consent was obtained from all patients.

#### Data Collection

The clinical manifestations, laboratory examination findings, BVAS, and data of treatment were collected through medical records. The laboratory examinations included blood cell count (WBC), hemoglobin (HB), platelet (PLT), neutrophils (N), lymphocytes (L), neutrophil–lymphocyte ratio (NLR), platelet lymphocyte ratio (PLR), mean platelet volume (MPV), red blood cell volume width-coefficient of variation (RDW-CV), red blood cell volume width-standard deviation (RDW-SD), erythrocyte sedimentation (ESR), C-reactive protein (CRP), complement 3 (C3), complement 4 (C4), glutamic-pyruvic transaminase (ALT), albumin (ALB), globulin (GLO), urea nitrogen (BUN), serum creatinine (Scr), uric acid (UA), hematuria, proteinuria, ANCA serotype including peripheral anti-neutrophil cytoplasmic antibody (p-ANCA), cytoplasmic anti-neutrophil cytoplasmic antibody (c-ANCA), myeloperoxidase (MPO), and protease 3 (PR3). The BVAS scores were evaluated by two experienced rheumatologists to determine the disease activity of patients with AAV after their diagnosis. In addition, the data on the treatments, such as glucocorticoid (GC), cyclophosphamide (CTX), mycophenolate mofetil (MMF), leflunomide, CD20 monoclonal antibody, and plasma exchange, were also collected. The number of patients with different conditions, such as deaths, lost visits, and survivors within 1 year, were counted via telephone follow-up.

#### Statistical Analysis

SPSS 26.0 software was used to compare the statistical differences and survival analysis of each index, and prism software was used to make the Kaplan–Meier (K-M) curves of patients with gender and AAV. All data between male and female patients with AAV were compared. Continuous data of normal distribution were expressed as mean standard deviation (*x* ± *SD*), and statistical differences between groups were compared using the *t*-test. Continuous data with skewed distribution were expressed with the median of the interquartile range (IQR), and statistical differences between the two groups were compared using the Mann–Whitney U test. Categorical data are expressed as ratios (%) and subjected to a chi-square test. According to the cut-off value of the ROC curve, the individual data were converted into binary classified variables, and the log-rank test and COX test were performed according to the log-likelihood ratio. In the COX model, forward stepwise regression was applied, and the default Wald's test was adopted. The standard *p*-value for variable elimination was 0.1 and the standard *p*-value for inclusion was 0.05. The results are expressed as hazard ratio and 95% confidence interval (CI). *P* < 0.05 was considered to indicate statistical significance.

### Meta-Analysis

#### Data Sources and Searches

This meta-analysis was designed and conducted following the PRISMA statement (Appendix 1). Databases such as China Biomedical Library (https://www.sinomed.ac.cn), CNKI (China National Knowledge Infrastructure, https://www.cnki.net), VIP database (https://www.cqvip.com), Wan fang database (https://wanfangdata.com.cn) in China, and Cochrane Library, Embase, PubMed, and Web of Science were all applied to search for articles published up to 12 July 2021 with the following terms: (“ANCA associated vasculitis” or “ANCA vasculitis” or “ANCA related vasculitis” or “AAV” or “GPA” or “MPA” or “EGPA”) AND (“Sex” or “Gender” or “Male” or “Female”) AND (“Prognosis” or “death” or “survival” or “mortality” or “death rate”) AND (“one year”) (Appendix 2). In addition, a manual search of eligible studies was conducted to determine other qualified studies. This search strategy was conducted twice in total (**Figure 2**).

#### Inclusion Criteria and Study Selection

The inclusion criteria were original articles on AAV (including randomized controlled studies, case-control studies, cohort studies, and cross-sectional studies), which analyzed the basic characteristics and with complete data, including country, region, data source, race, follow-up time, the overall number of people, number of male and female patients, and total death toll or number of deaths per sex. The exclusion criteria were case reports, case series reviews, meta-analysis guidelines, meeting abstractions, expert opinions, etc. Studies in which the follow-up time was not 1 year were excluded. The qualification of full-text articles was determined by four reviewers (QZ, BLC, QWY, and YSW), and ambiguous articles were checked by a fifth reviewer (YG). Reviewers BLC and QWY screened the titles and abstracts of the identified references and excluded articles unrelated to the topic of interest. Reviewer FL, XX, and YG conducted a comprehensive review of related articles.

#### Data Extraction and Quality Assessment

Reviewers (QZ, BLC, QWY, YSW, and YG) independently extracted the data for each included study and resolved any differences through a discussion. The following variables were obtained: author, publication year, data source, research type, the total number of patients with AAV, number of male and female patients, number of deaths and survivors, number of deaths per sex, and follow-up time. All data included in the study were extracted in standard form. Case-control studies were assessed using the Newcastle-Ottawa Scale (NOS). For a total score of nine points, studies that scored 0–3 points, 4–6 points, and 7–9 points were considered low-quality, medium-quality, and high-quality studies, respectively (23).

#### Meta-Analysis

Review Manager 5.3 software was used for meta-analysis, and Stata14.0 software was used for Egger's test and sensitivity analysis. Dichotomous data are expressed as odds ratio (OR) and 95% CI, and continuous data were expressed as standard mean difference and 95% CI. *P* < 0.05 was considered statistically significant. The chi-square test and *I*^2^ statistic were used to assess the heterogeneity of published studies. For studies with significant heterogeneity (*I*^2^>50%, *P* < 0.05), the random-effects model was used; otherwise, the fixed-effects model was employed. Funnel-plot and Egger's test were used to estimate the impact of possible publication bias (24). Subgroup analyses were further refined if heterogeneity existed.

## Results

### Results of the Single-Center Retrospective Analysis

#### Clinical Characteristics of Patients With AAV

A total of 84 patients with AAV initially diagnosed were included in this study, including 33 female and 51 male patients, with an average age of 56.6 years and a disease course of 5 months. The baseline characteristics of the patients are listed in Table 1. MPA was the most commonly noted disease, followed by GPA and EGPA (85.7% and 9.5 vs. 4.8%). Non-specific symptoms such as fever, fatigue, and weight loss were the most common (75%), followed by the involvement of the lungs (50%), kidneys (45.2%), joints and muscles (32.1%), ear, nose, and throat (7.1%), skin (4.8%), nervous system (4.8%), mucous membrane and eye (1.2%), and cardiovascular system (1.2%). As for therapeutic agents, the majority of the patients were treated with GC combined with CTX, followed by hydroxychloroquine. A few of the patients underwent treatment with MMF, leflunomide, or CD20 monoclonal antibody.

**Table 1:** Basic characteristics of patients with AAV and comparison between different genders.

	**Total (*N* = 84)**	**Group**		
		**Male (*N* = 51)**	**Female (*N* = 33)**	***P*-value**
Age (Y)	56.6 ± 13.5	57.9 ± 10.5	52.2 ± 16.7	0.005^*^
Disease duration (M)	5.0 ± 7.1	3.3 ± 5.1	7.5 ± 8.8	0.008^*^
**Mortality rate (** ***n*** **, %)**				
3-month	4 (4.8)	4 (7.8)	0 (0)	0.105
6-month	12 (14.3)	10 (19.6)	2 (6.1)	0.056
12-month	14 (16.7)	12 (23.5)	2 (6.1)	0.024^*^
**Classification of diseases (** ***n*** **, %)**				
GPA	8 (9.5)	5 (9.8)	3 (9.1)	0.913
MPA	72 (85.7)	42 (82.4)	30 (90.9)	0.118
EGPA	4 (4.8)	4 (7.8)	0 (0)	0.042^*^
**Types of ANCA (** ***n*** **, %)**				
p-ANCA	58 (69)	31 (60.8)	27 (81.8)	0.227
c-ANCA	6 (7.1)	4 (7.8)	2 (6.1)	0.596
MPO	74 (88.1)	46 (90.2)	28 (84.8)	0.311
PR3	11 (13.3)	8 (15.7)	3 (9.1)	0.353
**Organ involvement (** ***n*** **, %)**				
Lung	42 (50)	28 (54.9)	14 (42.4)	0.264
Kidney	38 (45.2)	20 (39.2)	18 (54.5)	0.168
Skin	4 (4.8)	2 (3.8)	2 (6.1)	0.657
Mucosa and eye	1 (1.2)	1 (2.0)	0 (0)	0.316
Ear, nose, throat	6 (7.1)	4 (7.8)	2 (6.1)	0.754
Cardiovascular	1 (1.2)	0 (0.0)	1 (3.0)	0.170
Nervous system	4 (4.8)	2 (3.9)	2 (6.1)	0.657
Joint and muscle	27 (32.1)	16 (31.4)	11 (33.3)	0.851
Non-specific symptoms	63 (75)	35 (68.6)	28 (84.8)	0.094
**Lab data (median, IQR)**				
WBC (10^9^/L)	9.3 [6.0–10.8]	10.2 [6.6–12.8]	8.0 [5.2–10.2]	0.044^*^
HB (g/l)	86 [72.3–96.5]	88 [76–98]	81.9 [69–89.5]	0.046^*^
PLT (10^9^/L)	278.7 [181–351]	280.4 [198–351]	276.2 [177–339]	0.689
N (10^9^/L)	7.4 [4.2–9.1]	8.2 [4.5–10.2]	6.2 [3.3–8.5]	0.048^*^
L (10^9^/L)	1.3 [0.9–1.6]	1.3 [0.9–1.7]	1.2 [0.8–1.5]	0.513
NLR	7.2 [3.0–5.5]	7.5 [3.6–8.8]	6.7 [2.7–8.8]	0.165
PLR	266.7 [144.3–347.5]	254.4 [141.0–345.9]	285.6 [149.8–382.6]	0.724
MPV (fL)	10.3 [9.4–11.0]	10.4 [9.4–11.1]	10.2 [9.5–11.0]	0.706
RDW-CV (%)	14.4 [12.9–15.6]	14.3 [12.7–15.5]	14.6 [13.2–15.6]	0.463
RDW-SD (fL)	45.9 [41.7–49.0]	45.8 [41.7–49.0]	45.9 [41.3-49.2]	0.818
ESR (mm/h)	69.1 [34–99.5]	77.2 [56–101.3]	56.8 [26–93.8]	0.03^*^
CRP (mg/l)	46.3 [6.49–69.3]	55.4 [8.4–84.6]	27.5 [26–46]	0.019^*^
C3 (g/l)	0.9 [0.7–1.0]	0.8 [0.7–1.0]	0.9 [0.9–1.0]	0.781
C4 (g/l)	0.2 [0.2-0.3]	0.2 [0.2–0.3]	0.2 [0.2–0.3]	0.735
AL (u/l)	22.7 [7.7-26.6]	27.0 [10.6–27.3]	15.7 [5.2–18.8]	0.013^*^
ALB (g/l)	29.3 [25–33.7]	28.2 [10.6–27.2]	31.1 [26.8–30.9]	0.016^*^
GLB (g/l)	31.5 [25.9–36.7]	31.4 [26.8–37.4]	31.7 [25.3–36.6]	0.848
TB (umol/l)	7.1 [4.3–8.3]	7.3 [4.1–9.1]	6.8 [4.3–8.2]	0.907
Scr (umol/l)	378.7 [126.2-528.2]	402.7 [196.6–389.9]	343.1 [80.5–559.8]	0.152
BUN (mmol/l)	17.5 [9.1–23.9]	20.0 [10.7–28.9]	13.6 [6.4–18.0]	0.013^*^
UA (ummol/l)	396.5 [300.2–483]	418.1 [330.1–517.7]	363.3 [239.4–454.9]	0.069
Hematuria (*n*, %)	76 (90.5)	45 (88.2)	31 (93.9)	0.518
Proteinuria (*n*, %)	65 (77.4)	40 (78.4)	25 (75.8)	0.559
BVAS	12 [9.5-−14.5]	12 [10–15]	10 [9-12.75]	0.008^*^
**Treatment after diagnosis (** ***n*** **, %)**				
Different doses of GC				
<0.5 mg/kg	2 (2.4)	2 (3.9)	0 (0)	0.149
0.5–1 mg/kg	23 (27.4)	16 (31.4)	7 (21.2)	0.267
>1 kg/mg	55 (65.5)	30 (58.8)	25 (75.8)	0.140
CTX	54 (64.3)	34 (66.7)	20 (60.6)	0.571
MMF	8 (9.5)	1 (2.0)	7 (21.2)	0.079
Plasma exchange	22 (26.2)	12 (23.5)	10 (30.3)	0.490
CD20 monoclonal antibody	2 (2.4)	1 (2.0)	1 (3.3)	0.756
Single drug or combination drugs				
1 drug	18 (21.4)	11 (21.6)	7 (21.2)	0.969
2 drugs	41 (48.8)	24 (47.1)	17 (51.5)	0.690
3 drugs	14 (16.7)	7 (13.7)	7 (21.2)	0.369
≥4 drugs	11 (13.1)	9 (17.6)	2 (6.1)	0.107

*Y, years; M, months; %, percentage; GPA, Granulomatous vasculitis; MPA, Microscopic vasculitis; EGPA, Eosinophilic granulomatous polyangiitis; c-ANCA, Cytoplasmic anti-neutrophil cytoplasmic antibody; p-ANCA, Perinuclear anti-neutrophilic cytoplasmic antibody; PR3, Protease 3; MPO, Myeloperoxidase; IQR, Interquartile range; WBC, White blood cell; HB, Hemoglobin; PLT, Platelet; N, Neutrophils; L, Lymphocytes; NLR, Neutrophil-lymphocyte ratio; PLR, Platelet lymphocyte ratio; MPV, Mean platelet volume; RDW-CV, Red blood cell volume width-variation of red blood cells; RDW-SD, Red blood cell volume distribution width-standard deviation; ESR, Erythrocyte sedimentation; CRP, C-reactive protein; C3, Complement 3; C4, Complement 4; ALT, Glutamic-pyruvic transaminase; sALB, Albumin; GLO, Globulin; TBIL, Total bilirubin; BUN, Urea nitrogen; Scr, Serum creatinine; UA, Uric acid; BVAS, Birmingham Vasculitis Activity Score; GC, Glucocorticoid; CTX, Cyclophosphamide; MMF, Mycophenolate mofetil*.

* *P < 0.05*.

Table 1 also displays baseline characteristics stratified by sex. The mean onset age of female patients with AAV was 52.2 years and for male patients, it was 57.9 years, and the mean disease duration before diagnosis was 7.5 months for female patients and 3.3 months for male patients with a significant difference (*P* < 0.05). The rate of organ involvement between men and women showed no statistical difference, but BVAS was higher in men than in women (12 vs. 10; *P* = 0.008). EGPA seemed more common in male patients (7.8 vs. 0%; *P* = 0.042). The laboratory indicators, including WBC, HB, N, ESR, CRP, BUN, and ALT, were all significantly higher in men, whereas ALB was lower than in women (*P* < 0.05; Table 1). There were no significant differences between the two groups concerning the indices of PLT, L, NLR, PLR, MPV, RDW-CV, RDW-SD, C3, C4, GLO, DBIL, Scr, UA, the ratio of hematuria and proteinuria, the usage ratio of therapeutic drugs including GC, CTX, MMF, plasma exchange and CD20 monoclonal antibody, and the number of combined therapeutic drugs.

#### Analysis of 1-Year Survival Rate and Risk Factors

**Figure 1:**
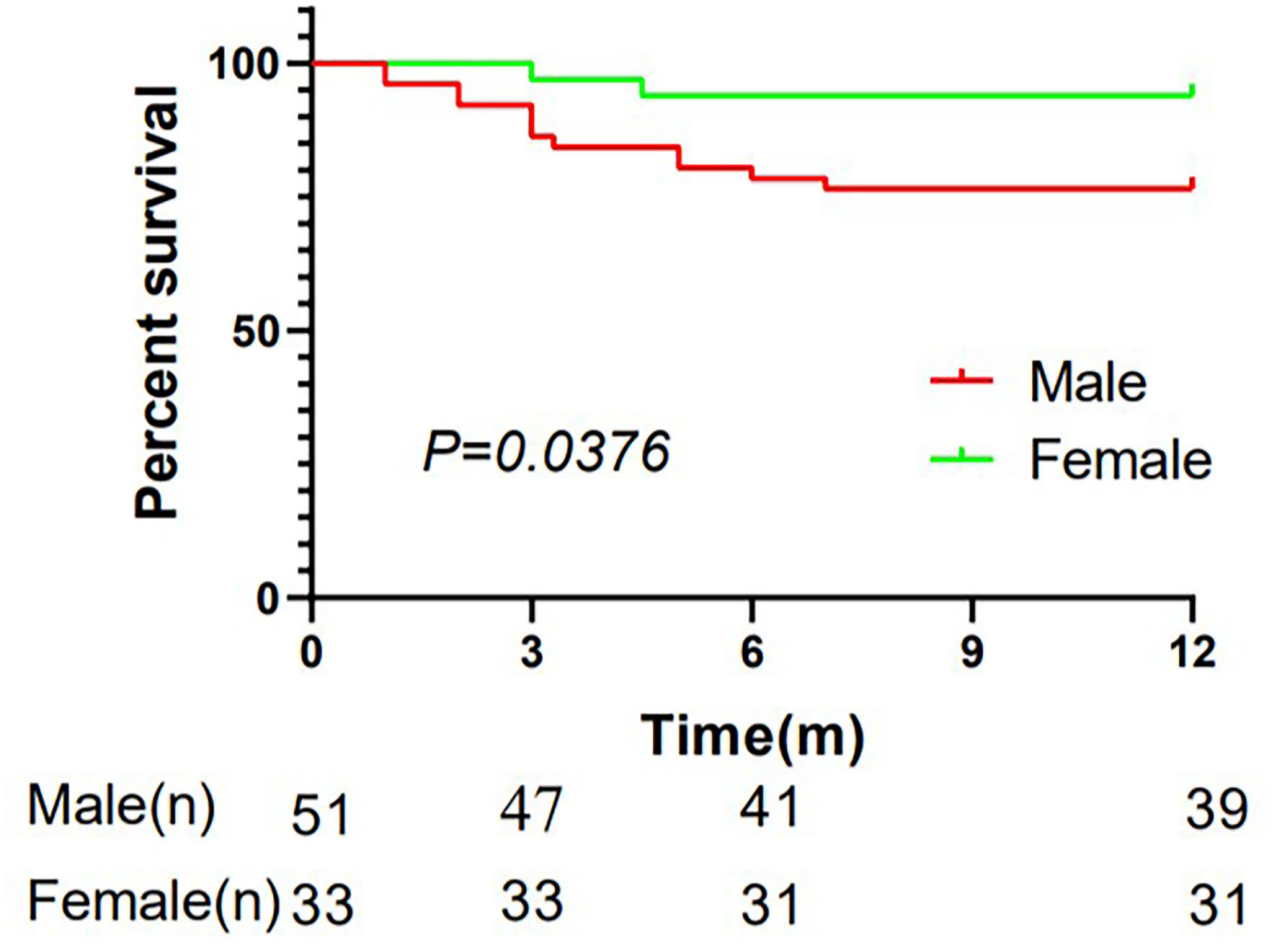
Kaplan–Meier plots demonstrating the mortality ratio of patients with AAV in males and females in the 1-year follow-up. 84 patients were included and 70 survived in the first year after diagnosis with 39 males and 31 females. The Kaplan-Meier (K-M) survival curves indicated that the 1-year mortality ratio was higher in male patients than in female patients (*P* = 0.0376). m, months.

All patients underwent telephone follow-up. A total of 14 people died (12 men and 2 women) and 70 survived in the 1st year after diagnosis. During 0–3 months follow-up, there were four deaths in the male group and none in the female group. During 3–6 months follow-up, there were six deaths in the male group and two in the female group. During 6–12 months follow-up, there were two deaths in the male group and none in the female group. There was no significant difference between the 3- or 6-month mortality ratio of male and female patients with AAV, whereas the mortality ratio of male patients with AAV was significantly higher in the 1st year than female patients (*P* < 0.05; Table 1). The K–M survival curves indicated that the 1-year mortality ratio was higher in male patients than in female patients (*P* = 0.0376; Figure 1).

Univariate regression analysis was conducted to analyze the risk factors of 1-year mortality in patients with AAV. Gender, routine blood indices (WBC, PLT, N, NLR, MPV, and RDW-CV/SD), CRP, liver function (TBIL), renal function (BUN, UA, Scr), the ratio of proteinuria, the BVAS, and combined two drugs for treatment were risk factors for 1-year mortality in patients with AAV. However, only male (OR = 5.41; 95% CI 1.19-24.59), elevated Scr (OR = 4.67; 95% CI 1.10-19.92), increased PLT (OR = 7.0; 95% CI 1.42-34.57), augmented NLR (OR = 15.87; 95% CI 1.48-170.15), and raised RDW-CV (OR = 3.27; 95% CI 1.14–9.38) were independent risk factors for 1-year mortality in patients with AAV(*P* < 0.05; Table 2).

**Table 2:** Factors related to 1-year mortality ratio in patients with AAV according to univariate regression and multivariate regression.

	**Univariate regression**		**Multivariate regression**	
	**OR**	***P*-value**	**OR [95%CI]**	***P*-value**
**Basic features**				
Gender(M/F)	5.01	0.025^*^	5.41 [1.19-24.59]	0.029^*^
Age	4.78	0.096		
Course of disease	2.76	0.096		
**Classification of diseases**				
GPA	0.20	0.652		
MPA	0.00	0.978		
EGPA	0.16	0.689		
**Types of ANCA**				
p-ANCA	0.13	0.719		
c-ANCA	0.01	0.92		
MPO	0.36	0.548		
PR3	0.00	0.978		
**Organ involvement**				
Lung	1.27	0.684		
Kidney	2.33	0.124		
Skin	2.56	0.442		
Mucosa and eye	0.00	1.000		
Ear, nose, throat	0.35	0.401		
Cardiovascular	0.67	1.00		
Nervous system	0.00	0.999		
Joint and muscle	1.67	0.367		
Non-specific symptoms	1.49	0.53		
**Laboratory indicators**				
WBC	14.63	0.000^*^	2.35 [0.46–11.88]	0.303
HB	1.19	0.276		
PLT	6.32	0.012^*^	7.00 [1.42–34.57]	0.017^*^
N	14.64	0.000^*^	2.35 [0.46–11.88]	0.303
L	0.58	0.448		
NLR	7.03	0.008^*^	15.87 [1.48–170.15]	0.022^*^
PLR	0.00	0.984		
MPV	10.72	0.001^*^	2.28 [0.38-13.55]	0.367
RDW-CV	4.26	0.039^*^	3.27 [1.14–9.38]	0.028^*^
RDW-SD	16.86	0.000^*^	2.73 [0.29–25.92]	0.383
ESR	1.72	0.19		
CRP	10.72	0.001^*^	2.28 [0.38–13.55]	0.367
C3	1.62	0.204		
C4	0.33	0.567		
ALT	2.93	0.087		
ALB	0.08	0.773		
GLB	3.46	0.063		
TBIL	6.07	0.014^*^	2.86 [0.77–10.67]	0.117
Scr	5.61	0.018^*^	4.67 [1.10–19.92]	0.037^*^
BUN	28.61	0.000^*^	5.17 [0.27–99.91]	0.277
UA	5.85	0.016^*^	1.85 [0.48–7.09]	0.372
Albuminuria	3.87	0.049^*^	0.35 [0.12–1.05]	0.062
Hematuria	0.12	0.727		
BVAS	5.26	0.022^*^	1.57 [0.50–4.97]	0.444
**Treatment after diagnosis**				
Different doses of GC				
<0.5mg/kg	0.396	0.529		
0.5–1mg/kg	0.417	0.518		
>1kg/mg	0.166	0.684		
CTX	2.012	0.156		
MMF	0.192	0.661		
Plasma exchange	0.386	0.535		
CD20 monoclonal antibody	0.404	0.525		
Single drug or combination drugs				
1 drug	0.98	0.323		
2 drugs	4.441	0.035^*^	3.38 [0.73–15.67]	0.12
3 drugs	1.232	0.267		
≥4 drugs	0.672	0.412		

*M/F, Males/Females; OR, Odds ratio; CI, Confidence interval; GPA, Granulomatous vasculitis; MPA, Microscopic vasculitis; EGPA, Eosinophilic granulomatous polyangiitis; c-ANCA, Cytoplasmic anti-neutrophil cytoplasmic antibody; p-ANCA, Perinuclear anti-neutrophilic cytoplasmic antibody; PR3, Protease 3; MPO, Myeloperoxidase; WBC, White blood cell; HB, Hemoglobin; PLT, Platelet; N, Neutrophils; L, Lymphocytes; NLR, Neutrophil-lymphocyte ratio; PLR, Platelet lymphocyte ratio; MPV, Mean platelet volume; RDW-CV, Red blood cell volume width-variation of red blood cells; RDW-SD, Red blood cell volume distribution width-standard deviation; ESR, Erythrocyte sedimentation; CRP, C-reactive protein; C3, Complement 3; C4: Complement 4; ALT, Glutamic-pyruvic transaminase; ALB, Albumin; GLO, Globulin; TBIL, Total bilirubin; BUN, Urea nitrogen; Scr, Serum creatinine; UA, Uric acid; BVAS, Birmingham Vasculitis Activity Score; GC, Glucocorticoid; CTX, Cyclophosphamide; MMF, Mycophenolate mofetil;*

* *P < 0.05*.

### Results of the Meta-Analysis

#### Study Selection

**Figure 2:**
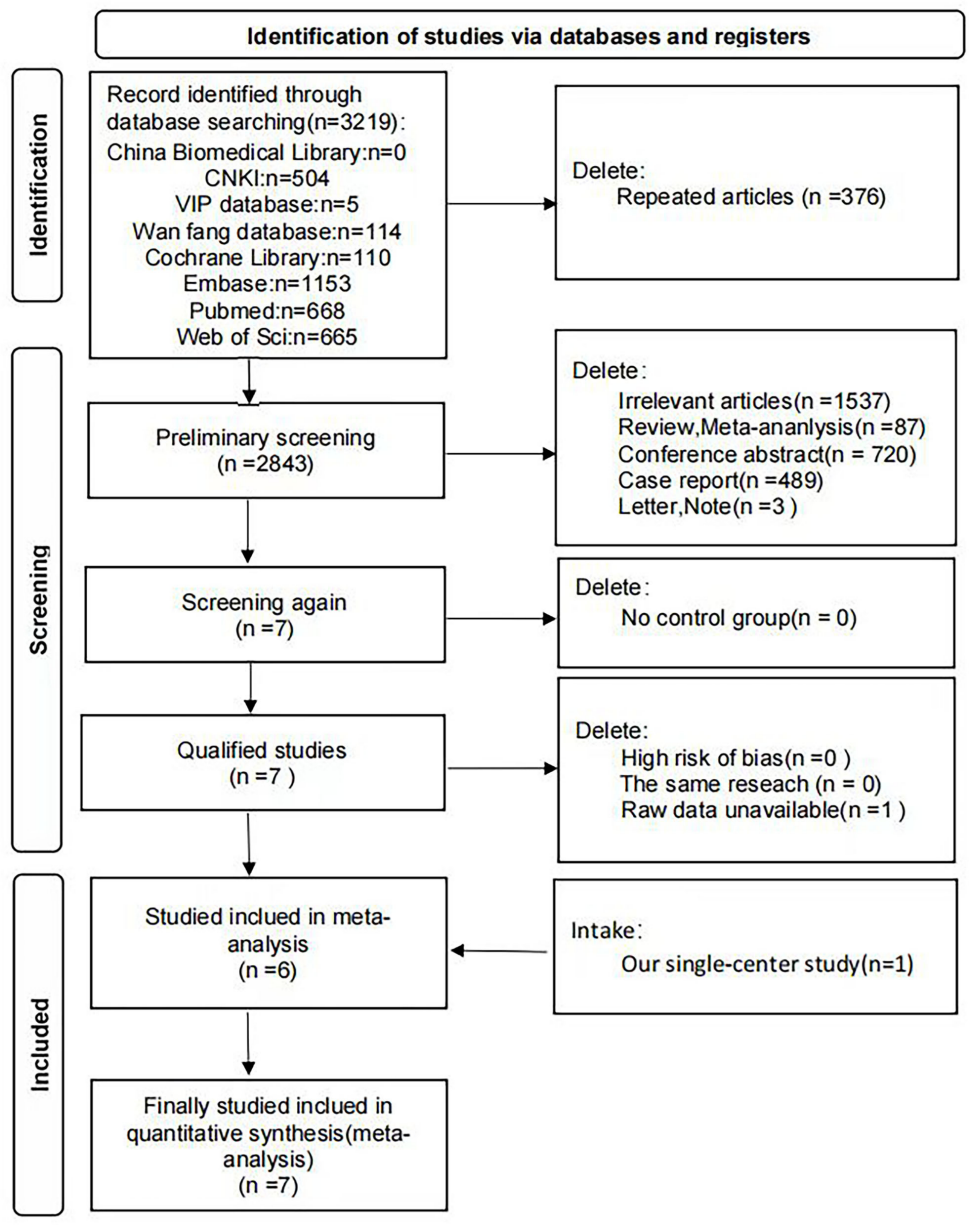
Flow chart of document screening for meta-analysis. Flow chart presenting the process of study selection for meta-analysis. Seven literatures including our single-center study were included finally.

The flow chart for study selection in the meta-analysis is shown in Figure 2. The initial search generated 3,219 studies, of which 0 were from the China Biomedical Library, 504 were from CNKI, five were from the VIP database, 114 were from the Wan fang database in China, and 110 were from Cochrane, 1,153 were from Embase, 668 were from PubMed, and 665 were from Web of Science. After deleting 376 duplicate articles, 2,843 articles were included in the preliminary screening, among which 1,537 articles were irrelevant to this study, 87 meta-analyses or systematic literature reviews, 720 conference abstracts, 489 case reports, and 3 letters or notes were excluded. After excluding articles that did not meet the inclusion criteria, 7 articles were considered qualified, but 1 of them was also excluded for lacking adequate data after failing to contact the author. Thus, six articles from the databases were included. In addition, considering our respective study also met the inclusion criteria, a total of seven studies were applied for meta-analysis finally (Figure 2).

#### Characteristics and Quality of the Included Studies

Table 3 listed the characteristics of the included studies. All were case-control studies conducted up to 12 July 2021, of which three studies were from Asia and 4 from Europe (14, 17, 19, 25–27). The diagnostic inclusion and exclusion criteria were reported in all the studies. A total of 1,136 patients with AAV were included, and men accounted for 51.32%. The total number of deaths was 210, with 126 being male (60%). According to the NOS, case-control studies included in the meta-analysis were evaluated. The NOS score was six, which indicated the case-control studies were of medium quality (Table 3).

**Table 3:** Main characteristics of included studies in the meta-analysis.

**References**	**Nation**	**Region**	**Data source**	**Design**	**No. of study (*n*)**	**Age (Y) (median, IQR)**	**M/F (*n*, %)**	**Death of M/F (*n*)**	**Quality score method**	**Quality assessment**	**Quality scale**
Slot et al. (17)	Holland	Europe	Single center	Case-control	85	56 [39–73]	55/30 (64.7, 35.3)	18/2	NOS	6	Moderate
Takala et al. (25)	Finland	Europe	Single center	Case-control	492	NS	243/249 (49.4, 50.6)	45/39	NOS	6	Moderate
Ono et al. (26)	Japan	Asia	Multi-center	Case-control	79	NS	36/43 (45.6, 54.4)	4/7	NOS	6	Moderate
Abe et al. (19)	Japan	Asia	Single center	Case-control	52	73.2 [62.4–84]	24/28 (46.2, 53.8)	9/9	NOS	6	Moderate
Heijl et al. (14)	Sweden	Europe	Single center	Case-control	195	69 [55–77]	97/98 (49.7, 50.3)	23/11	NOS	6	Moderate
Titeca-Beauport et al. (27)	France	Europe	Multi-center	Case-control	149	72.7 [68.5–76.8]	77/72 (51.7, 68.3)	15/14	NOS	6	Moderate
Zhu et al. 2021^*^	China	Asia	Single center	Case-control	84	56.6 [43.1–70.1]	51/33 (60.7, 39.3)	12/2	NOS	6	Moderate

*n, number; Y, years; IQR, Interquartile range; M/F, Male/Female; NS, Not search; NOS, the New Castle-Ottawa Scale*.

* *Data of our single-center retrospective analysis*.

#### Meta-Analysis and Sensitivity Analysis

**Figure 3:**
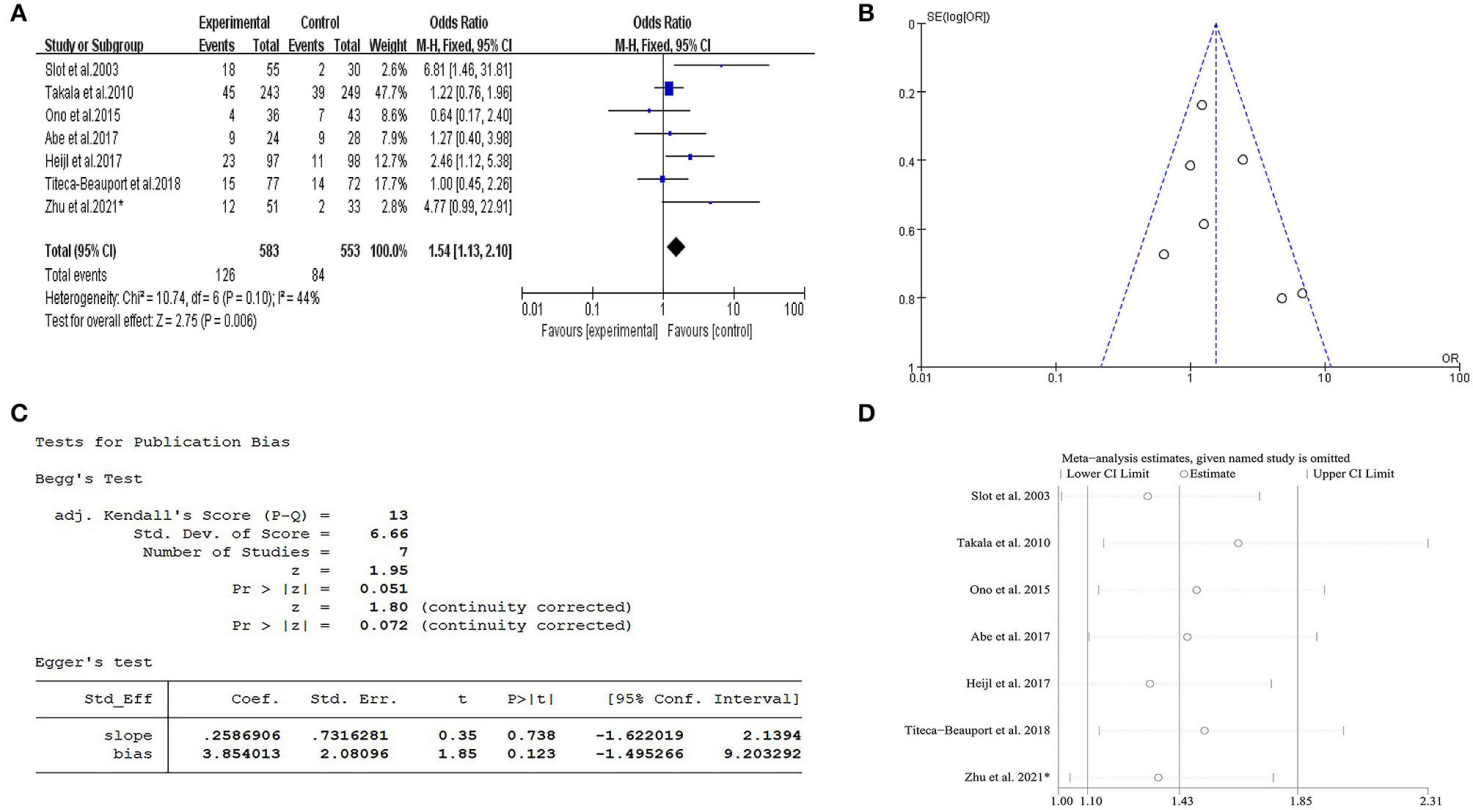
Meta-analysis about gender vs. 1-year mortality in AAV. **(A)** The forest map showed male was a risk factor for 1-year mortality of patients with AAV with OR value of 1.54 (95% CI: 1.13-−2.10; *P* = 0.006) and no significant heterogeneity (*I*^2^= 44%, *P* = 0.1). **(B)** Funnel plot indicated there was no publication bias. **(C)** Egger test also indicated no publication bias (*P* > 0.05). **(D)** Sensitivity analysis indicated that there was no significant change in the overall effect magnitude after removal of either study, and the results were robust and reliable. ^*^ Data of our single-center retrospective analysis.

The forest map showed male was a risk factor for 1-year mortality of patients with AAV with OR value of 1.54 (95% CI: 1.13–2.10; *P* = 0.006) and no significant heterogeneity (*I*^2^ = 44%, *P* = 0.1) (Figure 3A). Therefore, the fixed-effects model was used and subgroup analyses were not further refined. Subsequently, sensitivity analysis of the above research suggested that the research findings were reliable and robust (Figure 3D).

#### Publication Bias

A funnel plot and Egger's test were performed to evaluate the publication bias of this meta-analysis. The *P*-value of Egger's test was 0.123 (*P* > 0.05), which indicated there was no publication bias (Figure 3C). In this study, funnel plot shapes were found to be symmetrical (Figure 3B).

## Discussion

Anti-neutrophil cytoplasmic antibody (ANCA) associated vasculitis is a rare disease and only a few studies are available on the relationship between gender and prognosis. To the best of our knowledge, this study was the first one to explore the relationship between gender and 1-year mortality of AAV in the population of China. This single-center retrospective study demonstrated that gender was related to the short-term prognosis of patients with AAV in southern China, with a 1-year mortality ratio significantly higher in men than in women. In addition, our meta-analysis of seven studies also showed this significant correlation. This may provide a reliable predictor for clinicians to judge the prognosis and select active treatment for patients with AAV.

Patients with AAV generally have the highest risk of acute and severe injury within 1 year of onset (28), and the all-cause mortality of male patients is higher than that of female patients in terms of long-term survival in Korea (20). In our study, the number of EGPA and GPA patients was too small compared with MPA, so we did not compare differences between the three subgroups, but we mainly aimed to compare differences between males and females (Table 1). Under this premise, we found there were statistical differences in the age of onset, the course of the disease, the proportion of EGPA, BVAS, WBC, HB, N, ESR, CRP, ALT, ALB, BUN, and 1-year mortality ratio between male and female patients with AAV.

A multicenter study in 2019 reported that male patients with AAV more often suffered from respiratory and kidney damage at an early stage, which made them seek medical help for treatment earlier than female patients (29), and this was consistent with our conclusion that male patients had a shorter course of the disease. Epidemiological data on EGPA are scarce, accounting for only 10–20% of AAV cases (30). Retrospective studies in Peru showed a significant reduction in female vs. male EGPA (1: 4) (31), which was consistent with the population distribution in our study.

A higher WBC count on initial treatment was noted in male patients with AAV with higher BVAS in our study. Cornec D et al. reported that among patients with AAV treated with rituximab, male patients had lower B cell count and lower BVAS than females (32), and naive B-lymphopenia may be a biomarker of disease activity in AAV (33). These indicated that WBC count may be associated with the disease activity of male patients with AAV. There also was a statistical difference in HB between male patients with AAV and females in our study (88 vs. 81.9 g/L). Anemia was reported to be a common complication in patients with AAV, and HB <75 g/L seemed significantly correlated with the prognosis in patients with AAV (34). Although both male patients and females suffered moderate anemia (HB>75 g/L), whether it contributed to the poor prognosis of male patients or just represented the severity of the disease requires more data to determine.

A specific index for detecting liver function is ALT. It has been reported that in a healthy adolescent population, females have a negative relationship with ALT while males have a positive relationship, but no study has suggested the same relationship in adults (35). Besides, no research has confirmed that ALT is related to the prognosis of patients with AAV. Therefore, although there was a significant statistical difference in ALT between the two groups in this study, considering that the level of ALT was normal in both male and female groups, further research is needed to confirm whether this difference is of clinical significance. Low ALB was negatively correlated with CRP and ESR and was a good indicator for disease monitoring in AAV (36). ALB was lower in men than in women in our study, while ESR, CRP, and BVAS were higher in men than in women, which highlighted the possible correlation between low ALB, elevated ESR, CRP, and high disease activity in patients with AAV. A similar study also showed low ALB was associated with the disease severity and prognosis of myeloperoxidase-ANCA-associated glomerulonephritis (MPO-ANCA-GN) (37). But no studies have confirmed an association of lower ALB with 1-year mortality in patients with AAV, and further research is needed.

We found that the 1-year mortality rate of men was higher than that of women, with a significant statistical difference (*P* < 0.05). Among the 14 dead people, 12 were men and 2 women. Similar results were discovered by Caroline et al. in Sweden that among seven patients who died from vasculitis, six were men and only one was female (14), and the reason for death was whether the curative effect of drugs was not good, or the disease progressed rapidly. We thought the main reason for death in this study was the disease progression and deterioration, for we explicitly excluded other comorbidities which may affect the mortality rate of the AAV patient at the beginning.

Given the above differences between male and female patients in our study, we wondered whether gender was an independent risk factor for poor short-term prognosis in patients with AAV, so further data was analyzed. It is surprising to find that male gender was indeed a risk factor for 1-year mortality in patients with AAV in the regression analysis. The K–M curve also showed that the 1-year mortality ratio was significantly higher in male patients than in female patients with prolongation (*P* = 0.0376).

Except for male patients, elevated Scr, increased NLR, augmented PLT, and raised RDW-CV were also found to be poor prognostic factors in patients with AAV in our study, and this might provide a simpler and more convenient means for clinicians to evaluate the prognosis of patients during follow-up. In this study, elevated Scr was identified as a poor prognostic factor for patients with AAV, which was consistent with other research (29, 38). Neutrophils trigger autoimmune diseases (39). ANCA can stimulate neutrophils to release neutrophil extracellular traps containing autoantigens, and lead patients with AAV to have autoimmune responses to these components (40). The decrease of lymphocytes is related to the low recurrence rate of the disease (41). Recently studies indicated that NLR, the ratio of neutrophils to lymphocytes, was positively correlated with the poor diagnosis of AAV. We also found that NLR was an independent risk factor for the 1-year mortality of patients with AAV. The predictive role of NLR had been speculated to be explained by a negative correlation between NLR and C3 serum levels (42), whereas a decrease in C3 level was associated with a poor renal prognosis and patient outcome (43, 44). In addition, NLR also played a positive role in renal damage, and a higher baseline NLR led to a worse renal prognosis (45). RDW is a routine measurement of the heterogeneity of circulating red blood cell size and is clinically used to distinguish different types of anemia, especially iron-deficiency anemia and chronic anemia (46). Kim et al. found that RDW≥15.4% at diagnosis might increase the risk of severe GPA and predict refractory disease type (46), and this might be related to the presence of a large number of pro-inflammatory factors in patients with AAV during disease activity, while pro-inflammatory factors were associated with the development of anemia in various diseases (47). Here for the first time, we demonstrated that elevated RDW was an independent risk factor for patients with AAV, but the specific mechanism needed further investigation. We also found that an elevated PLT was an independent risk factor for patients with AAV; previous research showed that PLT count was significantly higher in patients with AAV with an active disease state than in those with a remission disease state (48), but relevant data were limited.

Among all the risk factors found in this study, the controversial relationship between gender and 1-year mortality in patients with AAV was our interest and focus as well. Hence, a systemic review and meta-analysis were conducted to confirm whether the male gender was related to the short-term prognosis of patients with AAV all over the world. We found that a total of seven studies from Asia and Europe previously covered the relationship between gender and its prognosis in patients with AAV, including our single-center study. The final forest map results of our meta-analysis indicated that the male gender was a risk factor for 1-year mortality in patients with AAV and the risk of death was 1.54 times higher in male patients, which was the same as our findings in retrospective analysis. The sensitivity analysis also proved that the result of the meta-analysis was reliable. Together, we believed this provided a higher level of evidence-based evidence for the effect of male gender on the short-term prognosis of patients with AAV and a tool for early prognosis prediction for clinicians.

There were some limitations to this study. First, patients were included from a single center and were inpatients in a large general hospital whose disease state was generally severe. If conditions permit, patients with different disease activities and patients who come from the community should be investigated, together with a larger population to accurately determine the influence of gender on mortality in patients with AAV in China. Second, considering that AAV is a rare case, and we cannot obtain more data to investigate the certain relationship between gender and prognosis of MPA, GPA, and EGPA, respectively, we hope to confirm their correlations in the near future. Finally, the increased RDW-CV and PLT were both found to be prognostic risk factors for patients with AAV in our study, but given the total number of enrolled patients and information from other studies, further investigations should be performed to clarify their relationship with the short-term prognosis of patients with AAV.

## Conclusion

Significant sex-specific differences were found in patients with AAV in Southern China. Male, elevated Scr, NLR, PLT, and RDW-CV were poor short-term prognostic factors for patients with AAV in the retrospective study. Among them, we clarified that male sex was a risk factor for 1-year mortality in patients with AAV by further meta-analysis. Clinicians should pay more attention to the mortality risk of male patients with AAV in the early stage, and intensive and careful management should be taken.

## Data Availability Statement

The raw data supporting the conclusions of this article will be made available by the authors, without undue reservation.

## Author Contributions

Material preparation, data collection, and analysis were performed by QZ, BC, QY, YW, and YG. The first draft of the manuscript was written by QZ and YG. The manuscript was critically revised by FL, XX, and YG. All authors commented on previous versions of the manuscript, read and approved the final manuscript, and contributed to the concept and design of the study.

## Funding

This study was supported by the National Natural Science Foundation of China (No. 81701622), Natural Science Foundation of Hunan province (No. 2021JJ30934), Hunan Provincial Health Committee 225 Talent Project (2019-196), Educational Fund of Hunan Provincial Finance Department (2021-22-2050205), and Natural Science Foundation of Changsha (No. kq2202409).

## Conflict of Interest

The authors declare that the research was conducted in the absence of any commercial or financial relationships that could be construed as a potential conflict of interest.

## Publisher's Note

All claims expressed in this article are solely those of the authors and do not necessarily represent those of their affiliated organizations, or those of the publisher, the editors and the reviewers. Any product that may be evaluated in this article, or claim that may be made by its manufacturer, is not guaranteed or endorsed by the publisher.
