# Supplementary material for: Relationship Between Gender and 1-Year Mortality in ANCA-Associated Vasculitis Patients: A Single-Center Retrospective Analysis and Meta-Analysis
Source: Front Med (Lausanne). 2022 Jul 13;9:945011. doi: 10.3389/fmed.2022.945011 (PMC9326069; doi:10.3389/fmed.2022.945011)
Supplement: Supplementary file 1 [file Data_Sheet_1.docx]

| **Section and Topic** | **Item #** | **Checklist item** | **Location where item is reported** |
| --- | --- | --- | --- |
| **TITLE** | | |  |
| Title | 1a | Identify the report as a systematic review; | Title |
|  | 1b | Report an informative title that provides key information about the main objective or question | Title |
| **ABSTRACT** | | |  |
| Abstract | 2 | See the PRISMA 2020 for Abstracts checklist. | Abstract |
| **INTRODUCTION** | | |  |
| Rationale | 3a | Describe the current state of knowledge and its uncertainties; | Introduction |
|  | 3b | Articulate why it is important to do the review. | Introduction |
| Objectives | 4 | Provide an explicit statement of questions being addressed with reference to participants, interventions, comparisons, outcomes, and study design (PICOS). | Introduction |
| **METHODS** | | |  |
| Eligibility criteria | 5a | Specify all study characteristics used to decide whether a study was eligible for inclusion in the review, that is, components described in the PICO framework or one of its variants, and other characteristics, such as eligible study design(s) and setting(s), and minimum duration of follow-up; | Methods and materials |
|  | 5b | Specify eligibility criteria with regard to report characteristics, such as year of dissemination, giving rationale and report status (e.g., whether reports, such as unpublished manuscripts and conference abstracts, were eligible for inclusion); | Methods and materials |
|  | 5c | Clearly indicate if studies were ineligible because the outcomes of interest were not measured, or ineligible because the results for the outcome of interest were not reported. | Methods and materials |
| Information sources | 6 | Specify all databases, registers, websites, organisations, reference lists and other sources searched or consulted to identify studies. Specify the date when each source was last searched or consulted. | Methods and materials |
| Search strategy | 7 | Present the full search strategies for all databases, registers and websites, including any filters and limits used. | Methods and materials |
| Selection process | 8 | Specify the methods used to decide whether a study met the inclusion criteria of the review, including how many reviewers screened each record and each report retrieved, whether they worked independently, and if applicable, details of automation tools used in the process. | Methods and materials |
| Data collection process | 9 | Specify the methods used to collect data from reports, including how many reviewers collected data from each report, whether they worked independently, any processes for obtaining or confirming data from study investigators, and if applicable, details of automation tools used in the process. | Methods and materials |
| Data items | 10a | List and define all outcomes for which data were sought. Specify whether all results that were compatible with each outcome domain in each study were sought (e.g., for all measures, time points, analyses), and if not, the methods used to decide which results to collect. | Methods and materials |
|  | 10b | List and define all other variables for which data were sought (e.g., participant and intervention characteristic). Describe any assumptions made about any missing or unclear information. | Methods and materials |
| Study risk of bias assessment | 11 | Specify the tool(s) (and version) used to assess risk of bias in the included studies. | Methods and materials |
| Effect measures | 12 | Specify for each outcome the effect measure(s) (e.g., risk ratio, mean difference) used in the synthesis or presentation of results. | Methods and materials |
| Synthesis methods | 13a | Describe the processes used to decide which studies were eligible for each synthesis (e.g., tabulating the study intervention characteristics and comparing against the planned groups for each synthesis). | Methods and materials |
|  | 13b | Describe any methods required to prepare the data for presentation or synthesis, such as handling of missing summary statistics, or data conversions. | Methods and materials |
|  | 13c | Describe any methods used to tabulate or visually display results of individual studies and syntheses. | Methods and materials |
|  | 13d | Describe the model(s), method(s) to identify the presence and extent of statistical heterogeneity, and software package(s) used. | Methods and materials |
|  | 13e | Describe any methods used to explore possible causes of heterogeneity among study results (e.g. subgroup analysis, meta-regression). | Methods and materials |
|  | 13f | Describe any sensitivity analyses conducted to assess robustness of the synthesized results. | Methods and materials |
| Reporting bias assessment | 14 | Describe any methods used to assess risk of bias due to missing results in a synthesis (arising from reporting biases). | Methods and materials |
| Certainty assessment | 15 | Describe any methods used to assess certainty (or confidence) in the body of evidence for an outcome. | Methods and materials |
| **RESULTS** | | |  |
| Study selection | 16a | Describe the results of the search and selection process, from the number of records identified in the search to the number of studies included in the review, ideally using a flow diagram. | Results |
|  | 16b | Cite studies that might appear to meet the inclusion criteria, but which were excluded, and explain why they were excluded. | Results |
| Study characteristics | 17 | Cite each included study and present its characteristics. | Results |
| Risk of bias in studies | 18 | Present assessments of risk of bias for each included study. | Results |
| Results of individual studies | 19 | For all outcomes, present, for each study: (a) summary statistics for each group (where appropriate) and (b) an effect estimate and its precision (e.g. confidence/credible interval) | Results |
| Results of syntheses | 20a | For each synthesis, briefly summarise the characteristics and risk of bias among contributing studies. | Results |
|  | 20b | Present for each the summary estimate and its precision (e.g. confidence/credible interval) and measures of statistical heterogeneity. | Results |
|  | 20c | Present results of all investigations of possible causes of heterogeneity among study results. | Results |
|  | 20d | Present results of all sensitivity analyses conducted to assess the robustness of the synthesized results. | Results |
| Reporting biases | 21 | Present assessments of risk of bias due to missing results (arising from reporting biases) for each synthesis assessed. | Results |
| Certainty of evidence | 22 | Present assessments of confidence in the body of evidence for each outcome assessed. | Results |
| **DISCUSSION** | | |  |
| Discussion | 23a | Provide a general interpretation of the results in the context of other evidence. | Discussion |
|  | 23b | Discuss any limitations of the evidence included in the review. | Discussion |
|  | 23c | Discuss any limitations of the review processes used. | Discussion |
|  | 23d | Discuss implications of the results for practice, policy, and future research. | Discussion |
| **OTHER INFORMATION** | | |  |
| Registration and protocol | 24a | Provide registration information for the review, including register name and registration number. | Other information |
|  | 24b | Indicate where the review protocol can be accessed. | Other information |
|  | 24c | Describe and explain any amendments to information provided at registration or in the protocol. | Other information |
| Support | 25 | Describe sources of financial support for the review, and the role of the funders or sponsors in the review. | Other information |
| Competing interests | 26 | Declare any competing interests of review authors. | Other information |
| Availability of data, code and other materials | 27 | Report which of the following are publicly available and where they can be found: template data collection forms; data extracted from included studies; data used for all analyses; analytic code; any other materials used in the review. | Other information |
