# Supplementary material for: Relationship Between Gender and 1-Year Mortality in ANCA-Associated Vasculitis Patients: A Single-Center Retrospective Analysis and Meta-Analysis
Source: Front Med (Lausanne). 2022 Jul 13;9:945011. doi: 10.3389/fmed.2022.945011 (PMC9326069; doi:10.3389/fmed.2022.945011)
Supplement: Supplementary file 2 [file Data_Sheet_2.docx]

Search strategy of the electrical databases [performed on 12 July 2021]

| Database | Search strategy |
| --- | --- |
| China Biomedical Library | 1.ANCA associated vasculitis OR ANCA vasculitis OR ANCA related vasculitis OR AAV OR GPA OR MPA OR EGPA  2.Sex OR Gender OR Male OR Female  3.Prognosis OR death OR survival OR mortality OR deathrate  4.one year  5.(1AND 2 AND 3 AND 4) (MeSH)  6.Select studies of 5 |
| Cochrane | 1.ANCA associated vasculitis OR ANCA vasculitis OR ANCA related vasculitis OR AAV OR GPA OR MPA OR EGPA  2.Sex OR Gender OR Male OR Female  3.Prognosis OR death OR survival OR mortality OR deathrate  4.one year  5.(1 AND 2 AND 3 AND 4): ti, ab, kw  6.Select studies of 5 |
| CNKI | 1.ANCA associated vasculitis OR ANCA vasculitis OR ANCA related vasculitis OR AAV OR GPA OR MPA OR EGPA  2.Sex OR Gender OR Male OR Female  3.Prognosis OR death OR survival OR mortality OR deathrate  4.one year  5.(1 AND 2 AND 3 AND 4): ti, ab, kw  6.Select studies of 5 |
| Embase | 1.ANCA associated vasculitis OR ANCA vasculitis OR ANCA related vasculitis OR AAV OR GPA OR MPA OR EGPA  2.Sex OR Gender OR Male OR Female  3.Prognosis OR death OR survival OR mortality OR deathrate  4.one year  5.(1 AND 2 AND 3 AND 4): ti, ab  6.Select studies of 5 |
| Pubmed | 1.ANCA associated vasculitis OR ANCA vasculitis OR ANCA related vasculitis OR AAV OR GPA OR MPA OR EGPA  2.Sex OR Gender OR Male OR Female  3.Prognosis OR death OR survival OR mortality OR deathrate  4.one year  5.(1 AND 2 AND 3 AND 4) [Title/Abstract] [MeSH Terms]  6.Select studies of 5 |
| VIP database | 1.ANCA associated vasculitis OR ANCA vasculitis OR ANCA related vasculitis OR AAV OR GPA OR MPA OR EGPA  2.Sex OR Gender OR Male OR Female  3.Prognosis OR death OR survival OR mortality OR deathrate  4.one year  5.(1 AND 2 AND 3 AND 4): U, M, K  6.Select studies of 5 |
| Web of sci | 1.ANCA associated vasculitis OR ANCA vasculitis OR ANCA related vasculitis OR AAV OR GPA OR MPA OR EGPA  2.Sex OR Gender OR Male OR Female  3.Prognosis OR death OR survival OR mortality OR deathrate  4.one year  5.(1 AND 2 AND 3 AND 4): ti, ab  6.Select studies of 5 |
| Wanfang database | 1.ANCA associated vasculitis OR ANCA vasculitis OR ANCA related vasculitis OR AAV OR GPA OR MPA OR EGPA  2.Sex OR Gender OR Male OR Female  3.Prognosis OR death OR survival OR mortality OR deathrate  4.one year  5.(1 AND 2 AND 3 AND 4): ti, ab, kw  6.Select studies of 5 |
